# Surgical resection of a retroperitoneal liposarcoma producing insulin-like growth factor II: a case report

**DOI:** 10.1186/s40792-023-01589-9

**Published:** 2023-02-08

**Authors:** Noriyuki Nishiwaki, Yoshihiro Mikuriya, Fumiaki Takatsu, Ryoji Ochiai, Tomokazu Kakishita, Naruyuki Kobayashi, Takaya Kobatake, Shinji Hato, Norihiro Teramoto, Mototsugu Nagao, Izumi Fukuda, Koji Ohta

**Affiliations:** 1 Department of Gastroenterological Surgery, National Hospital Organization, Shikoku Cancer Center, 160, Ko, Minamiumemoto-machi, Matsuyama-shi, Ehime-Ken 791-0280 Japan; 2 Department of Pathology, National Hospital Organization, Shikoku Cancer Center, 160, Ko, Minamiumemoto-machi, Matsuyama-shi, Ehime-Ken 791-0280 Japan; 3 Department of Endocrinology, Metabolism and Nephrology, Graduate School of Medicine, Nippon Medical School, Bunkyo-ku, Tokyo, 113-8603 Japan

**Keywords:** Liposarcoma, Insulin-like growth factor, Hypoglycemia

## Abstract

**Background:**

Tumor-produced high molecular weight insulin-like growth factor-II (big insulin-like growth factor-II) is considered to cause non-islet cell tumor hypoglycemia. This paper presents a case of surgically resected retroperitoneal liposarcoma that produced big insulin-like growth factor-II.

**Case presentation:**

Here, we report the case of a 62-year-old woman who presented with an abdominal mass and hypoglycemia. Non-islet cell tumor hypoglycemia due to retroperitoneal liposarcoma was suspected. After complete resection of the tumor, the patient’s hypoglycemia improved and big insulin-like growth factor-II disappeared in the molecular weight analysis of serum insulin-like growth factor-II by western blotting. The patient had no tumor recurrence or reappearance of hypoglycemia 16 months after the operation without any adjuvant therapy.

**Conclusions:**

Although insulin-like growth factor-II-producing tumors are generally large and difficult to operate on, surgical resection is currently the most effective and only treatment; thus, it is essential to attempt resection aggressively.

## Background

Non-islet cell tumor hypoglycemia (NICTH) has been reported as the second major cause of spontaneous hypoglycemic attacks, next to insulinoma [1]. Tumor-produced high molecular weight insulin-like growth factor (IGF)-II (big IGF-II) is considered to cause hypoglycemia, and big IGF-II production by various tumors, such as hepatocellular carcinoma, gastric cancer, mesothelioma, hemangiopericytoma, leiomyosarcoma, and fibrous tumor, has been previously reported [2]. While there are few reports on IGF-II-producing tumors caused by liposarcoma, complete resection of IGF-II-producing liposarcoma has been rarely reported [3]. In this article, we report a case of liposarcoma in which an IGF-II-producing tumor was suspected based on preoperative imaging findings and hypoglycemic symptoms, and wherein the hypoglycemic symptoms improved after tumor resection.

## Case presentation

**Fig. 1:**
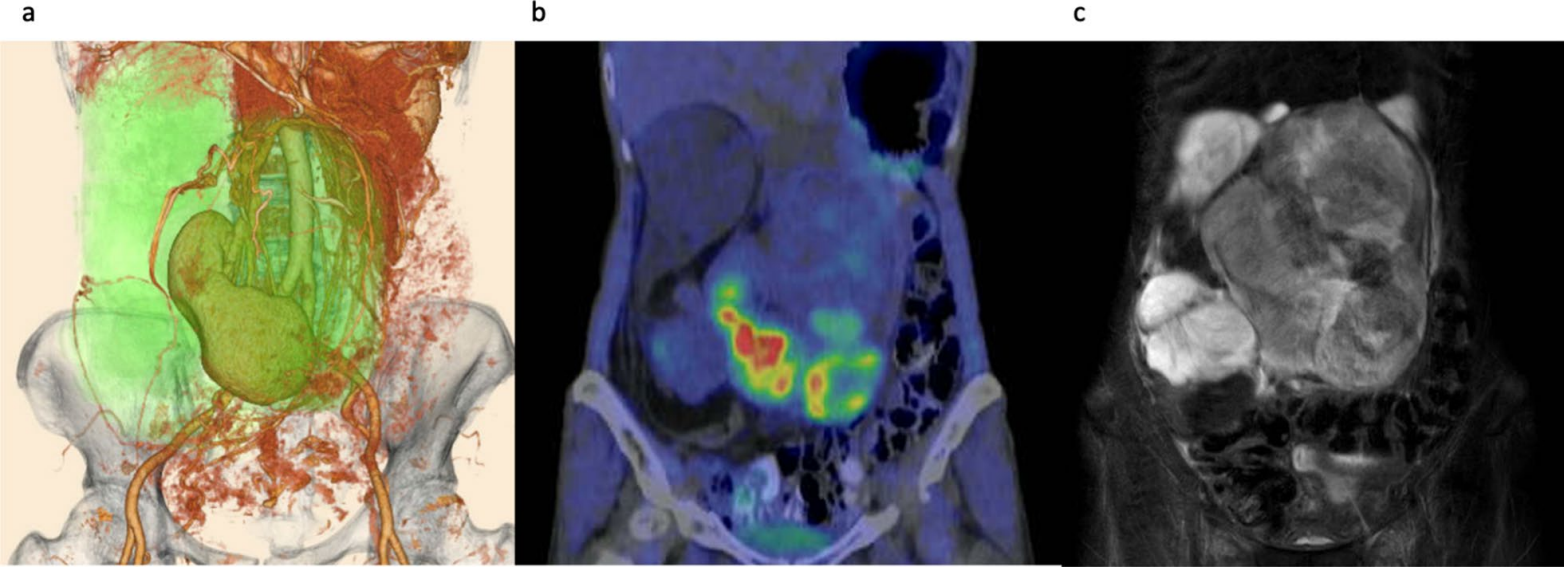
Preoperative image. Three-dimensionl image of computed tomography reconstruction revealed retroperitoneal huge tumor surrounding right kidney (green area) (**a**). Positron emission tomography–CT showed a mixture of areas with and without fluorodeoxyglucose accumulation (**b**). T2-weighted magnetic resonance imaging showed a mixture of fatty and myxoid components (**c**)

**Fig. 2:**
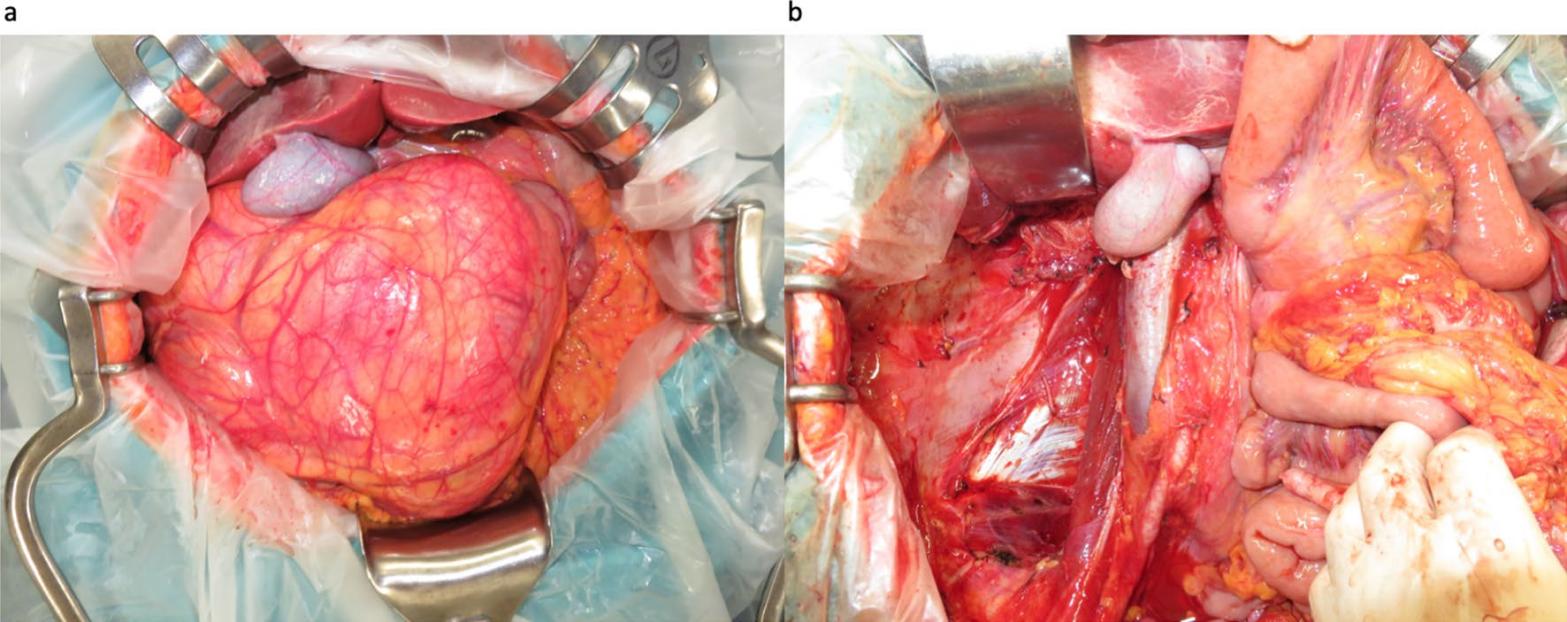
Intraoperative findings. The tumor was tightly adherent to the liver, duodenum, transverse colon, and inferior vena cava (**a**). The tumor, right kidney, and the fat in the right retroperitoneal space were completely excised (**b**)

**Fig. 3:**
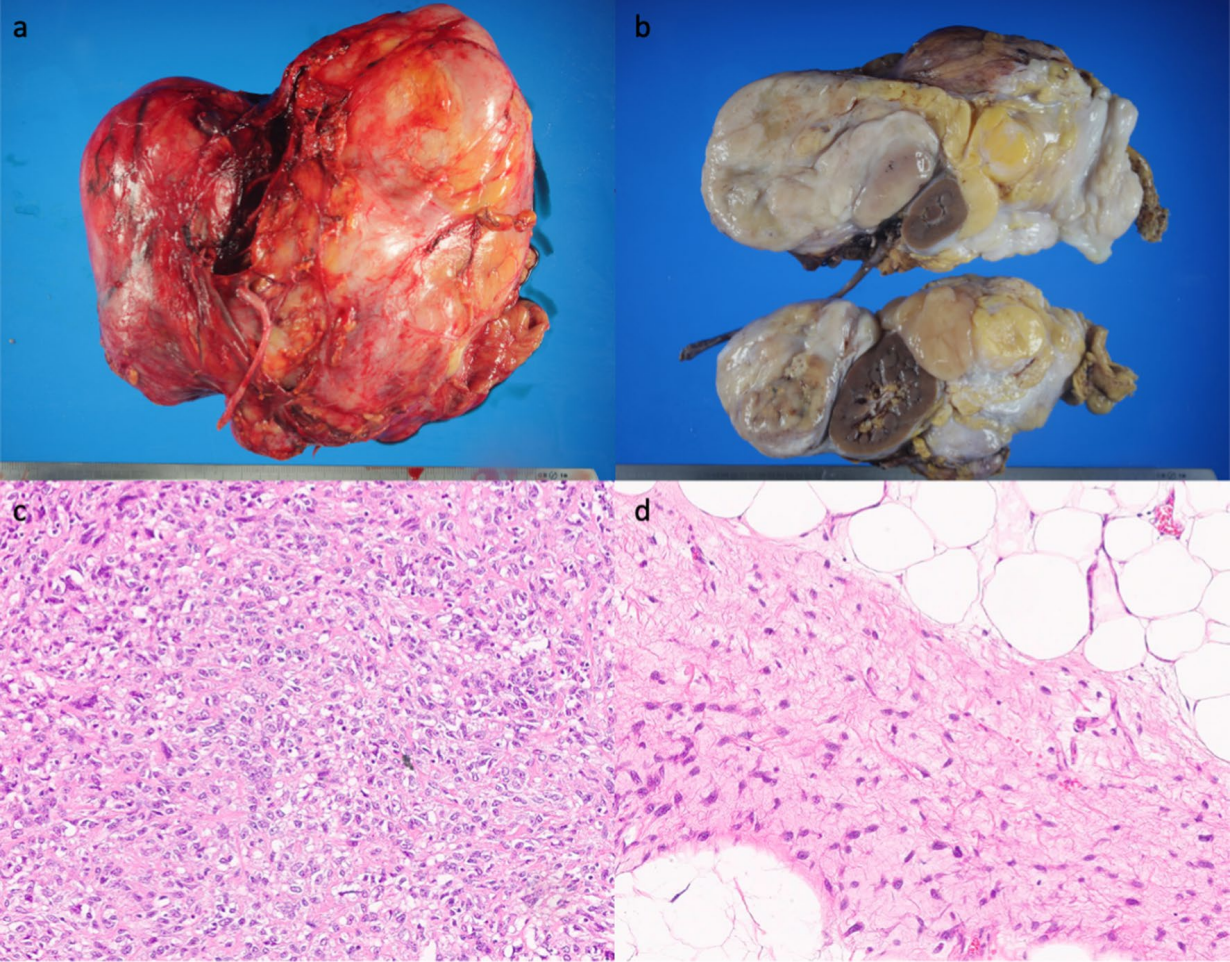
Macroscopic and microscopic findings of the tumor. The excised specimen was a lobulated mass measuring 27 × 27 × 13 cm and 2700 g (**a**). The right kidney was surrounded by the tumor (**b**). Proliferation of atypical short spindle or oval cells resembling high grade undifferentiated sarcoma (**c**). The presence of a well differentiated liposarcoma component, showing mature-appearing adipose tissue and fibrous bands with irregular nuclei (**d**)

**Fig. 4:**
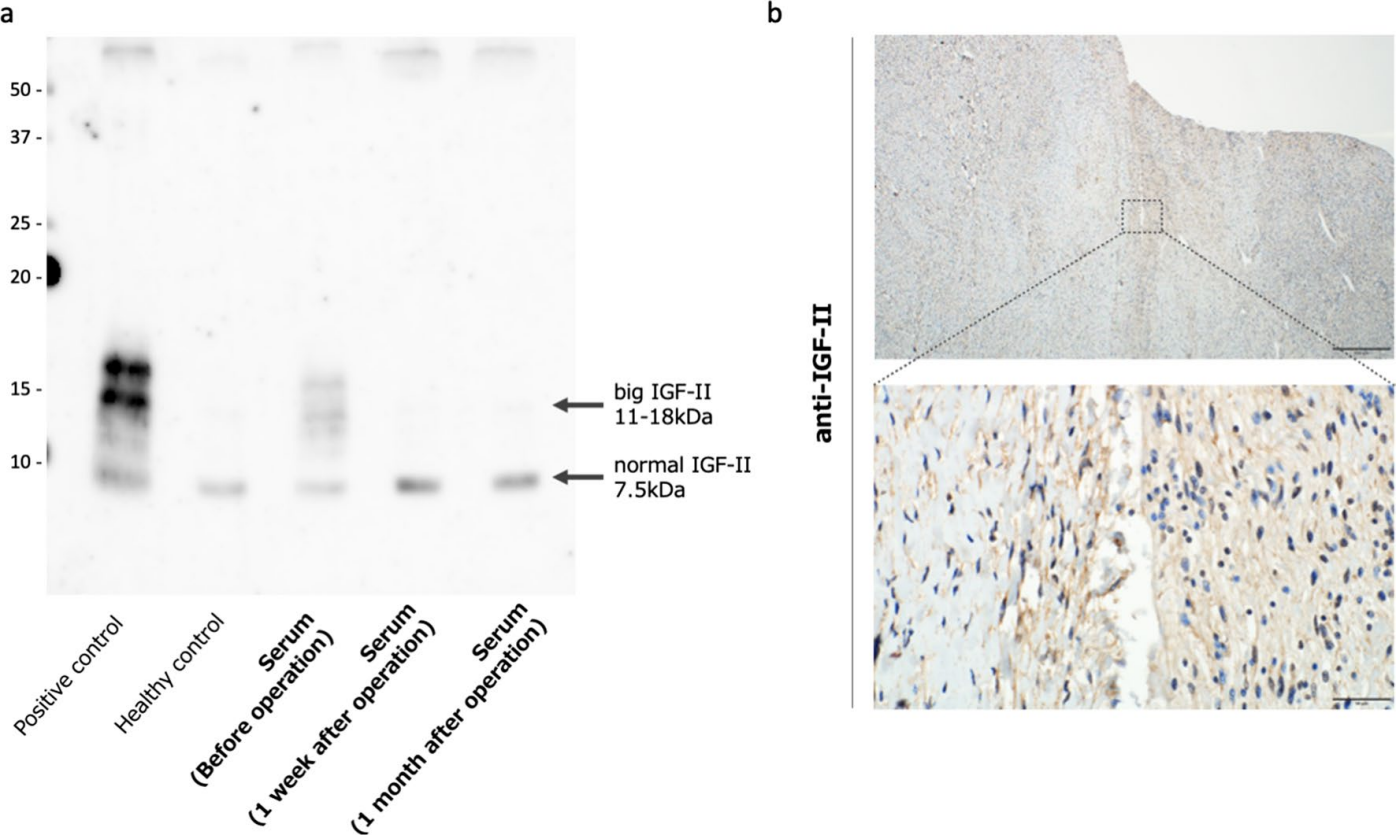
Western blot images showed that migrated bands were seen around 15 kDa (big IGF-II) in the serum sample before operation and disappeared after operation (**a**). Immunohistochemistry showed cytoplasm of the tumor cells was stained by IGF-II (**b**)

A 62-year-old woman presented to the hospital with the chief complaint of abdominal distension. Upon physical examination, a head-sized elastic soft mass became palpable in the midline of the abdomen. The patient was 158 cm tall, weighed 42 kg, had a body mass index (BMI) of 16.8, and had a markedly impaired appetite due to the pressure of the mass. Laboratory tests revealed severe hypoglycemia (2 mmol/L) as well as decreased levels of potassium (3.2 mmol/L), IGF-I (20 ng/mL), insulin (< 1.5 µIU/mL), and C-peptide (0.2 ng/mL). Although the patient did not exhibit the usual symptoms of hypoglycemia, a diet of 1800 kcal per day and supplementary snacks between meals and before sleep were necessary to avoid hypoglycemia. Computed tomography (CT) revealed a large retroperitoneal tumor (green area) surrounding the right kidney. The right kidney was deviated caudally medially due to tumor compression (Fig. 1a). Positron emission tomography–CT showed a mixture of areas with and without fluorodeoxyglucose accumulation (Fig. 1b). Magnetic resonance imaging showed a mixture of fatty and myxoid components (Fig. 1c). The patient had been preoperatively diagnosed to have NICTH due to liposarcoma and underwent retroperitoneal tumor resection. According to the intraoperative findings, the tumor was lumped with fat in the retroperitoneum, and the right kidney was completely inside the tumor. Moreover, the tumor was tightly adherent to the liver, duodenum, transverse colon, and inferior vena cava (Fig. 2a). We decided to perform a combined resection of the right kidney and carefully dissected the remaining organs to preserve them. Since the tumor was thought to have been derived from the fat in the retroperitoneum, the fat in the right retroperitoneal space was completely excised (Fig. 2b). The total operation time was 300 min, and the blood loss was 2095 mL, requiring an intraoperative transfusion of 8 units of red blood cells and 4 units of fresh frozen plasma. The excised specimen was a lobulated mass measuring 27 × 27 × 13 cm and 2700 g (Fig. 3a). Since the right kidney had been surrounded by the tumor, it displayed microscopic infiltration (Fig. 3b). The histological diagnosis was dedifferentiated liposarcoma, including partial areas of well-differentiated liposarcoma (Fig. 3c, d). According to the Federatin Nationale des Centres de Lutte Contre le Cancer grading system, the tumor was classified as grade 3 (a total score of 6:3 for dedifferentiated liposarcoma, 2 for 10–19 mitoses per 10 high-power fields, and 1 for < 50% tumor necrosis) and T4aN0M0 Stage IIIB (UICC 8^th^ TNM classification). The postoperative course was uneventful; the patient started eating on the second postoperative day and was discharged without complications on the 16th day. Postoperatively, her blood glucose level increased, and hypoglycemia was no longer observed. As for the IGF-II production in the tumor, western blotting (WB) of the serum showed that big IGF-II was observed before the surgery and it disappeared after the surgery (Fig. 4a). Immunohistochemistry (IHC) showed positive IGF-II in the tumor area (Fig. 4b). Results of the WB and IHC proved that big IGF-II had been produced by the tumor and decreased through tumor resection. The patient had no tumor recurrence or reappearance of hypoglycemia 16 months after the operation without any adjuvant therapy.

## Discussion

In this study, we reported a completely resected retroperitoneal liposarcoma that produced IGF-II. IGF-II production by the tumor was proven by the WB and IHC findings, and the patient’s hypoglycemic symptoms improved after the surgery, indicating that big IGF-II from the tumor had been related to the hypoglycemic symptoms.

The IGF system is regulated by three structurally similar ligands, IGF-I, IGF-II (IGFs), and insulin [4]. Although IGF-II plays important roles in fetal growth and development, IGF-II serum concentration rapidly declines after birth, with the role of IGF-II in adult cell functioning being relatively small [5, 6]. The structure of IGFs is similar to that of proinsulin. Although their potency is about 1/10 that of insulin, they elicit a hypoglycemic effect. IGF-II-producing NICTH cases have the common feature of big IGF-II with a molecular weight of 11–18 kDa being present in large amounts in the blood and tumors compared to the original 7.5 kDa IGF-II. The main body of big IGF-II is pro-IGF-IIE, wherein some amino acids of the E chain of pro-IGF-II, the precursor of IGF-II, remain. Hypoglycemia in IGF-II-producing NICTH is thought to be caused by an increase in tumor-derived free big IGF-II, and the big IGF-II is present in the blood as a binary complex of IGF-IGF binding protein that does not form an IGF-II ternary complex and can easily pass through the capillary wall to the cell surface receptor [7].

IGF-II is widely known to contribute to cell proliferation and tumor development in autocrine and paracrine manners. IGF-II expression is upregulated in several forms of cancer and the dysregulation of IGF-II expression is associated with cancer progression [8]. Specifically, IGF-II-producing tumors are usually large tumors, and Fukuda et al. reported that 70% of the patients in their study, which reviewed the clinical features of 78 cases of IGF-II producing NICTH, had tumors > 10 cm in size [9]. However, the characteristics of NICTH are still not fully understood. Although the clinical features of a large tumor with hypoglycemia lead to suspicions of IGF-II-producing NICTH, molecular weight analysis of serum IGF-II by WB is necessary for a definitive diagnosis, and this has only been performed in a limited number of research institutions. Recently, microRNA 483 family members are receiving attention as an alternative biomarker for diagnosing IGF-II-producing NICTH [10]. It is expected that the test will be available in more facilities and that the characteristics of IGF-II-producing NICTH will be clarified in the future.

Liposarcoma is one of the most common histologies of soft tissue sarcoma, representing 50% of all retroperitoneal sarcomas [11]. Well-differentiated and dedifferentiated liposarcomas are the most common type of retroperitoneal liposarcoma and the 5-year disease-specific survival in patients with dedifferentiated liposarcoma is 44%, compared to the 93% among those with pure well-differentiated liposarcoma. Since dedifferentiated liposarcoma has a low response to chemotherapy, surgery remains the mainstay treatment. Although the association between liposarcoma and IGF-II has been previously reported [12], there have been only few reports on the complete resection of IGF-II-producing liposarcoma. Among the ten previously reported cases of IGF-II-producing liposarcoma [3, 13, 14], complete resection was performed for only one case. This may be because some cases of retroperitoneal liposarcoma cannot be completely resected despite attempted surgery because of its rapid progression and frequent contact with vital organs [3].

Although first-line treatment for IGF-II-producing NICTH is complete tumor resection, the primary tumor is often large and complete resection is difficult. Some reports demonstrated that when the tumor is unresectable or when the operation results in an R2 resection, the administration of glucocorticoids and glucagon, chemoradiation of the primary tumor, and continuous infusion of glucose were effective [15–17]. However, these treatments only offer symptomatic relief and are not curative [18]. In our case, preoperative imaging showed that the tumor was in contact with multiple organs, including the duodenum, liver, transverse colon, gallbladder, pancreas, and right kidney, and that there was a possibility of a combined resection. As a result, it was detachable from most of the organs and only the right kidney underwent a combined resection due to tumor invasion. Although our follow-up period was short, the patient did not have tumor recurrence after the surgery. For retroperitoneal sarcoma, R2 resection is associated with significantly poor outcomes compared to R0 or R1 resection [19]. Although chemotherapy and radiation therapy could be used as adjuvant therapy for treating sarcomas, there is still no consensus regarding their efficacy [20, 21]. Liposarcomas, in particular, are resistant to those therapies and complete surgical resection is the most effective treatment. Therefore, careful dissection to preserve the organs and complete resection of the retroperitoneal adipose tissue without leaving any residue could be effective for rapid postoperative recovery and the prevention of recurrence.

## Conclusions

Here, we reported a case of surgically resected retroperitoneal liposarcoma that produced IGF-II in a patient with hypoglycemic symptoms. Although IGF-II-producing tumors are generally large and difficult to operate on, surgical resection is currently the most effective and only treatment; thus it is essential to attempt resection aggressively.

## Data Availability

The data supporting the conclusions are included in the article.

## Abbreviations

BMI: Body mass index
CT: Computed tomography
IGF: Insulin-like growth factor
IHC: Immunohistochemistry
NICTH: Non-islet cell tumor hypoglycemia
WB: Western blotting
